# Supplementary material for: Mapping social relevance in affective scenes: A large-scale multidimensional rating study
Source: PLoS One. 2026 Oct 8;21(10):e0331156. doi: 10.1371/journal.pone.0331156 (PMC13649022; doi:10.1371/journal.pone.0331156)
Supplement: Supplementary file 1 [file pone.0331156.s001.docx]

Supporting Information

Mapping Social Relevance in Affective Scenes:

A Large-Scale Multidimensional Rating Study

Vanessa Mitschke^1,2^, Francesco Grassi^1,2^, Alperen Doganer^1,2^, Anne Schacht^1,2*^

**Affiliations**
^1^ Department of Cognition, Emotion and Behavior, Institute of Psychology, University of Goettingen, Goettingen, Germany

^2^ CRC 1528 Cognition of Interaction, Goettingen, Germany

***Corresponding Author
E-mail:** [schacht@psych.uni-goettingen.de](mailto:schacht@psych.uni-goettingen.de) (AS)

Per-Image Dispersion Statistics

For each individual image, the standard deviation of each rating variable was determined across all participants. **Table S1** presents the median and range of the per-image standard deviation across all images.

Table S1. Median and range of the per-image standard deviation of each rating variable across all images.

|  | **Per-Image SD**, N = 296 |
| --- | --- |
| **Social Relevance** |  |
| Median | 1.87 |
| Min, Max | 0.87, 3.16 |
| **Pleasantness** |  |
| Median | 1.68 |
| Min, Max | 0.47, 2.61 |
| **Participation** |  |
| Median | 2.06 |
| Min, Max | 0.95, 2.82 |
| **Emotion Sharing ^1^** |  |
| Median | 1.98 |
| Min, Max | 0.76, 2.90 |
| **Action Sharing ^1^** |  |
| Median | 2.16 |
| Min, Max | 0.93, 3.20 |
| **Equality ^1^** |  |
| Median | 2.01 |
| Min, Max | 0.58, 3.27 |
| ^1^Only multiple-person images (N = 193) | |

Results of the Exploratory Correlation Analysis

Table S2. Semimatrix of d-correlations between ratings and objective features for single-person images.

|  | **Pleasantness** | **Participation** | **Face Visibility** | **Gaze Direction Viewer** | **N Faces** |
| --- | --- | --- | --- | --- | --- |
| Social Relevance | 0.95 | 0.29 | 0.18 | -0.01 | 0.36 |
| Pleasantness |  | 0.31 | 0.14 | 0.00 | 0.29 |
| Participation |  |  | 0.06 | 0.01 | 0.01 |
| Face Visibility |  |  |  | -0.02 | 0.77 |
| Gaze Direction Viewer |  |  |  |  | -0.01 |

Table S3. Semimatrix of d-correlations between ratings and objective features for multiple-person images.

|  | **Pleasantness** | **Participation** | **Emotion Sharing** | **Action Sharing** | **Equality** | **Face Visibility** | **Gaze Direction Viewer** | **Gaze Direction Within** | **N Faces** | **N People** | **People Group** |
| --- | --- | --- | --- | --- | --- | --- | --- | --- | --- | --- | --- |
| Social Relevance | 0.92 | -0.09 | 0.78 | 0.76 | 0.70 | 0.04 | -0.02 | 0.04 | 0.17 | 0.13 | 0.00 |
| Pleasantness |  | -0.04 | 0.76 | 0.70 | 0.70 | 0.04 | 0.00 | 0.02 | 0.11 | 0.07 | 0.00 |
| Participation |  |  | -0.06 | -0.02 | -0.11 | 0.02 | -0.02 | -0.03 | 0.09 | 0.30 | 0.06 |
| Emotion Sharing |  |  |  | 0.95 | 0.90 | 0.07 | -0.05 | 0.05 | 0.2 | 0.18 | 0.01 |
| Action Sharing |  |  |  |  | 0.81 | 0.08 | -0.04 | 0.03 | 0.21 | 0.21 | 0.01 |
| Equality |  |  |  |  |  | 0.05 | -0.04 | 0.07 | 0.21 | 0.17 | 0.00 |
| Face Visibility |  |  |  |  |  |  | 0.05 | -0.03 | 0.38 | -0.02 | 0.10 |
| Gaze Direction Viewer |  |  |  |  |  |  |  | -0.10 | 0.17 | 0.05 | 0.01 |
| Gaze Direction Within |  |  |  |  |  |  |  |  | -0.05 | 0.02 | 0.00 |
| N Face |  |  |  |  |  |  |  |  |  | 0.55 | 0.13 |
| N People |  |  |  |  |  |  |  |  |  |  | 0.41 |

Factor Analysis on Ratings of Engagement

Given the very strong correlation between emotion sharing, action sharing and equality, we conducted an Exploratory Factor Analysis (EFA) to investigate how well these three variables load onto a potential underlying latent factor. The analysis was conducted in R using function *fa* from package *psych* (version 2.5.3, Revelle, 2007).

Since we conducted EFA on three variables, we could only extract one latent factor. In fact, degrees of freedom in an EFA are calculated as:

$$\frac{1}{2}p\left( p+1 \right)-t$$

Where $p$ is the number of observed variables and $t$ the number of parameters being estimated (including both factor loadings and the unique variance). Calculating one factor from three observed variables means estimating three factor loadings and three respective unique variances ($t = 6$). Therefore, we end up with **zero degrees of freedom.** Trying to calculate even more factors would end up estimating more parameters than the data can support.

Please note that a consequence of having zero degrees of freedom, is that the model estimated here is **saturated**. The estimated factor will explain the shared variance among variables, but **there is no way to statistically test** if this factor solution is a good fit or not. Therefore, here we won’t report measures of model fit.

The results of the EFA are showed in (**Table S4**).

Table S4. Results of the EFA on the ratings of emotion sharing, action sharing and equality.

| **Rating Variable** | **Factor 1** | **Communality** | **Uniqueness** | **Complexity** |
| --- | --- | --- | --- | --- |
| Emotion Sharing | 1.00 | 1.00 | 0.00 | 1 |
| Action Sharing | 0.95 | 0.91 | 0.09 | 1 |
| Equality | 0.91 | 0.82 | 0.18 | 1 |

The EFA revealed that emotion sharing, action sharing and equality load quite clearly on a single factor explains a **very high portion of total variance** (91%). **A**ll three variables show **very large positive loadings** on the factor (> 0.90). **Compared to the other two variables, equality** contributes a slightly **less** to the **communal variance** (h^2^ = 0.82) and has a slightly **larger uniqueness** (u^2^ = 0.18).

Effect of Scene Pleasantness on Social Relevance

**Table S5** shows a summary of the LMM used to test the effect of scene pleasantness on social relevance, while controlling for scene participation, person number and gaze direction.

Table S5. Summary of the LMM regarding the effect of scene pleasantness on social relevance.

| **Predictors** | **R2** | **Estimate** | **std. Error** | **95% CI** | **t value** | **p** |
| --- | --- | --- | --- | --- | --- | --- |
|  | .43 |  |  |  |  |  |
| intercept |  | 5.23 | 0.12 | 4.99 5.47 |  | ^(1)^ |
| pleasantness^(2)^ |  | 1.63 | 0.04 | 1.55 1.71 | 41.37 | < .001 |
| participation^(2)^ |  | 0.21 | 0.03 | 0.16 0.26 | 7.80 | < .001 |
| person number (multiple) ^(3)^ |  | 0.78 | 0.12 | 0.55 1.00 | 6.49 | < .001 |
| gaze direction (averted) ^(4)^ |  | -0.25 | 0.13 | -0.50 0.01 | -1.96 | .052 |
| pleasantness^(2)^ * person number (multiple) ^(3)^ |  | -0.10 | 0.04 | -0.16 -0.03 | -2.72 | .006 |
| participation^(2)^ * person number (multiple) ^(3)^ |  | -0.16 | 0.03 | -0.22 -0.11 | -5.75 | < .001 |

(1) not shown because of being of very limited interpretability

(2) z-transformed to mean of zero and a standard deviation of one

(3) comparison with the reference level (single)

(4) comparison with the reference level (direct)

Interaction Effect of Scene Participation and Person Number of Social Relevance Rating.

The model also showed a significant interaction effect of participation and person number (*β* = -0.16, *SE* = 0.03, *p* < .001, 95% CI [-0.22, -0.11]). For single-person images, increased ratings of scene participation resulted in increased social relevance, while this effect was drastically reduced for multiple-person images (**Figure S1**).

**[S1 Fig about here]**

Figure S1. Social relevance as a function of scene participation, for single- and multiple-person images. Shaded areas represent 95% confidence intervals.

Effect of Engagement on Social Relevance

**Table S6** shows a summary of the LMM used to test the effect of engagement on social relevance, while controlling for scene participation and gaze direction.

Table S6. Summary of the LMM regarding the effect of engagement on social relevance.

| **Predictors** | **R2** | **Estimate** | **std. Error** | **95% CI** | **t value** | **p** |
| --- | --- | --- | --- | --- | --- | --- |
|  | .08 |  |  |  |  |  |
| intercept |  | 6.55 | 0.31 | 5.90 7.19 |  | ^(1)^ |
| engagement^(2)^ |  | 0.63 | 0.03 | 0.58 0.69 | 21.34 | < .001 |
| participation^(2)^ |  | 0.10 | 0.03 | 0.05 0.15 | 3.92 | < .001 |
| gaze direction (averted) ^(3)^ |  | -0.84 | 0.35 | -1.54 -0.11 | -2.43 | .017 |

(1) not shown because of being of very limited interpretability

(2) z-transformed to mean of zero and a standard deviation of one

(3) comparison with the reference level (direct)

Main Effects of Scene Participation and Gaze direction of Social Relevance Rating.

The model also showed a significant main effect of scene participation (*β* = 0.10, *SE* = 0.03, *p* < .001, 95% CI [0.05, 0.15]), with increased participation resulting in increased social relevance, and a main effect of gaze direction (*β* = -0.84, *SE* = 0.35, *p* = .017, 95% CI [-1.54, -0.11]), with lower ratings of social relevance for images with averted compared to direct gaze (**Figure S2**).

**[S2 Fig about here]**

Figure S2. Social relevance as a function of scene participation, for direct and averted gaze direction. Shaded areas represent 95% confidence intervals.

Moderation of Engagement Effects by Personality Traits

**Table S7** shows a summary of the LMM used to test the moderation effect of personality traits on the effect of engagement on social relevance.

Table S7. Summary of the LMM regarding the moderation by personality traits on the effects of engagement on social relevance.

| **Predictors** | **R2** | **Estimate** | **std. Error** | **95% CI** | **t value** | **p** |
| --- | --- | --- | --- | --- | --- | --- |
|  | .09 |  |  |  |  |  |
| intercept |  | 6.55 | 0.32 | 5.96 7.16 |  | ^(1)^ |
| engagement^(2)^ |  | 0.63 | 0.03 | 0.57 0.68 | 21.58 | < .001 |
| empathic concern^(2)^ |  | -0.00 | 0.03 | -0.07 0.06 | -0.08 | .940 |
| fantasy^(2)^ |  | 0.03 | 0.04 | -0.05 0.10 | 0.69 | .493 |
| personal distress^(2)^ |  | 0.01 | 0.04 | -0.06 0.08 | 0.26 | .791 |
| perspective taking^(2)^ |  | -0.00 | 0.04 | -0.07 0.06 | -0.13 | .896 |
| agreeableness^(2)^ |  | -0.00 | 0.04 | -0.08 0.08 | -0.05 | .955 |
| conscientiousness^(2)^ |  | 0.01 | 0.04 | -0.07 0.07 | 0.16 | .875 |
| extraversion^(2)^ |  | 0.00 | 0.04 | -0.08 0.08 | 0.12 | .906 |
| neuroticism^(2)^ |  | -0.01 | 0.04 | -0.08 0.07 | -0.23 | .816 |
| openness^(2)^ |  | 0.18 | 0.04 | 0.12 0.26 | 4.97 | < .001 |
| participation^(2)^ |  | 0.10 | 0.03 | 0.05 0.15 | 3.92 | < .001 |
| gaze direction (averted) ^(3)^ |  | -0.84 | 0.35 | -1.49 -0.17 | -2.43 | .017 |
| engagement^(2)^ * empathic concern^(2)^ |  | -0.02 | 0.02 | -0.06 0.02 | -0.89 | .381 |
| engagement^(2)^ * fantasy^(2)^ |  | 0.03 | 0.02 | -0.02 0.07 | 1.11 | .274 |
| engagement^(2)^ * personal distress^(2)^ |  | 0.03 | 0.02 | -0.01 0.08 | 1.40 | .171 |
| engagement^(2)^ * perspective taking^(2)^ |  | -0.03 | 0.02 | -0.08 0.02 | -1.33 | .189 |
| engagement^(2)^ * agreeableness^(2)^ |  | 0.03 | 0.02 | -0.02 0.07 | 1.22 | .230 |
| engagement^(2)^ * conscientiousness^(2)^ |  | -0.01 | 0.02 | -0.05 0.03 | -0.44 | .664 |
| engagement^(2)^ * extraversion^(2)^ |  | 0.06 | 0.02 | 0.01 0.11 | 2.57 | .012 |
| engagement^(2)^ * neuroticism^(2)^ |  | 0.02 | 0.02 | -0.03 0.07 | 0.77 | .450 |
| engagement^(2)^ * openness^(2)^ |  | 0.02 | 0.02 | -0.03 0.06 | 0.69 | .492 |

(1) not shown because of being of very limited interpretability

(2) z-transformed to mean of zero and a standard deviation of one

(3) comparison with the reference level (direct)
